# Agreement Between Apple Watch and Actical Step Counts in a Community Setting: Cross-Sectional Investigation From the Framingham Heart Study

**DOI:** 10.2196/54631

**Published:** 2024-07-24

**Authors:** Nicole L Spartano, Yuankai Zhang, Chunyu Liu, Ariel Chernofsky, Honghuang Lin, Ludovic Trinquart, Belinda Borrelli, Chathurangi H Pathiravasan, Vik Kheterpal, Christopher Nowak, Ramachandran S Vasan, Emelia J Benjamin, David D McManus, Joanne M Murabito

**Affiliations:** 1 Section of Endocrinology, Diabetes, Nutrition, and Weight Management Boston University Chobanian and Avedisian School of Medicine Boston, MA United States; 2 Boston University's and National Heart, Lung, and Blood Institute's Framingham Heart Study Framingham, MA United States; 3 Boston University School of Public Health Boston, MA United States; 4 University of Massachusetts Chan Medical School Worcester, MA United States; 5 Institute for Clinical Research and Health Policy Studies Tufts Medical Center Boston, MA United States; 6 Tufts Clinical and Translational Science Institute Tufts University Boston, MA United States; 7 Boston University Henry M. Goldman School of Dental Medicine Center for Behavioral Science Research Boston, MA United States; 8 Care Evolution Ann Arbor, MI United States; 9 Section of Preventive Medicine and Epidemiology, Department of Medicine Boston University Chobanian and Avedisian School of Medicine Boston, MA United States; 10 University of Texas School of Public Health and University of Texas Health Sciences Center San Antonio, TX United States; 11 Section of Cardiology, Department of Medicine Boston Medical Center, Boston University Chobanian and Avedisian School of Medicine Boston, MA United States; 12 Department of Epidemiology Boston University School of Public Health Boston, MA United States; 13 Department of Medicine University of Massachusetts Chan Medical School Worcester, MA United States; 14 Department of Population and Quantitative Health Sciences University of Massachusetts Chan Medical School Worcester, MA United States; 15 Section of General Internal Medicine, Department of Medicine Boston University Chobanian and Avedisian School of Medicine and Boston Medical Center Boston, MA United States

**Keywords:** accelerometer, mobile health, mHealth, wearable device, fitness tracker, physical activity, mobile phone, Apple Watch, step counts, Framingham Heart Study, Actical, digital health, tracker, wearable, wearables

## Abstract

**Background:**

Step counting is comparable among many research-grade and consumer-grade accelerometers in laboratory settings.

**Objective:**

The purpose of this study was to compare the agreement between Actical and Apple Watch step-counting in a community setting.

**Methods:**

Among Third Generation Framingham Heart Study participants (N=3486), we examined the agreement of step-counting between those who wore a consumer-grade accelerometer (Apple Watch Series 0) and a research-grade accelerometer (Actical) on the same days. Secondarily, we examined the agreement during each hour when both devices were worn to account for differences in wear time between devices.

**Results:**

We studied 523 participants (n=3223 person-days, mean age 51.7, SD 8.9 years; women: n=298, 57.0%). Between devices, we observed modest correlation (intraclass correlation [ICC] 0.56, 95% CI 0.54-0.59), poor continuous agreement (29.7%, n=957 of days having steps counts with ≤15% difference), a mean difference of 499 steps per day higher count by Actical, and wide limits of agreement, roughly ±9000 steps per day. However, devices showed stronger agreement in identifying who meets various steps per day thresholds (eg, at 8000 steps per day, kappa coefficient=0.49), for which devices were concordant for 74.8% (n=391) of participants. In secondary analyses, in the hours during which both devices were worn (n=456 participants, n=18,760 person-hours), the correlation was much stronger (ICC 0.86, 95% CI 0.85-0.86), but continuous agreement remained poor (27.3%, n=5115 of hours having step counts with ≤15% difference) between devices and was slightly worse for those with mobility limitations or obesity.

**Conclusions:**

Our investigation suggests poor overall agreement between steps counted by the Actical device and those counted by the Apple Watch device, with stronger agreement in discriminating who meets certain step thresholds. The impact of these challenges may be minimized if accelerometers are used by individuals to determine whether they are meeting physical activity guidelines or tracking step counts. It is also possible that some of the limitations of these older accelerometers may be improved in newer devices.

## Introduction

Physical inactivity is an important risk factor for many chronic diseases including obesity, diabetes mellitus, hypertension, cardiovascular disease, and dementia [1]. The 2018 Physical Activity Guidelines for Americans recommend 150 minutes of moderate to vigorous physical activity (MVPA) or more per week [1]. Despite many known benefits of physical activity, many Americans do not meet the Physical Activity Guidelines, the proportion of Americans meeting these guidelines changes drastically depending on whether physical activity levels are measured using accelerometers or self-report. Guideline achievement has been estimated to be as low as 15% of Americans using accelerometry in a nationally representative sample, or as high as 66% using self-reported data in the same individuals [2,3]. Furthermore, experts have expressed concern over whether these guidelines are appropriate and attainable, especially in older adults or those with mobility limitations [1,4,5].

Walking is a central component of physical activity and public health promotion efforts [6]. Public health messages focused on daily step counts may be a more appropriate target for achieving recommended amounts of physical activity in adults [6], which might have even more significance in older populations and those who have low MVPA levels. We are in a new paradigm in health care, in which 69% of US adults report tracking at least 1 health metric [7,8], including millions of individuals who track their steps using wearable accelerometer devices that are available commercially [9]. Despite the longstanding use of step counting in public health interventions [10], the Physical Activity Guidelines Committee has not yet created recommendations for the number of daily steps to target as a goal for health promotion [1]. The primary reason for this lack of step count guidelines has been a lack of evidence, but meta-analyses conducted from large cohort studies have recently reported that step count is associated with a lower risk of death and chronic disease [11,12]. Many accelerometers and pedometers have been validated to accurately count steps in the laboratory setting [13-15], but a remaining concern is that it is unclear how the number of steps reported in studies using research-grade accelerometers compares to steps counted by consumer-grade wearable devices used by the public living in the community (ie, the free-living setting).

During a recent Framingham Heart Study (FHS) exam cycle, physical activity was measured using both a consumer or mobile health device (Apple Watch) and a research-grade accelerometer (Actical) at the same time in the same individuals. The purpose of this investigation was to assess the agreement between Apple Watch and Actical-derived daily step count in free-living environments. We primarily assessed whether step count agreed when devices were worn on the same day, even if wear times differed, because we acknowledge that wear time and behavior may differ when participants wear different devices in the real world. We secondarily assessed whether agreement differed when devices were worn for the same hour block and whether agreement differed by age, sex, height, BMI, or those with mobility disabilities. This report will enable a better interpretation of the Apple Watch’s daily step count for research studies and consumers using these devices.

## Methods

### Study Cohort

The FHS Third Generation-based (Gen 3) cohort was recruited in 2002-2005 (n=4095) [16], and consisted mostly of grandchildren of the Original FHS cohort [17], who were largely individuals of European descent. The Gen 3–based examinations also included spouses of the Original cohort’s offspring (New Offspring Spouses [NOS], n=103) who were not already included in the Offspring (Generation 2) cohort and included a multiethnic Omni Group 2 (n=410). Participants from these cohorts have been examined every 6-8 years.

**Figure 1:**
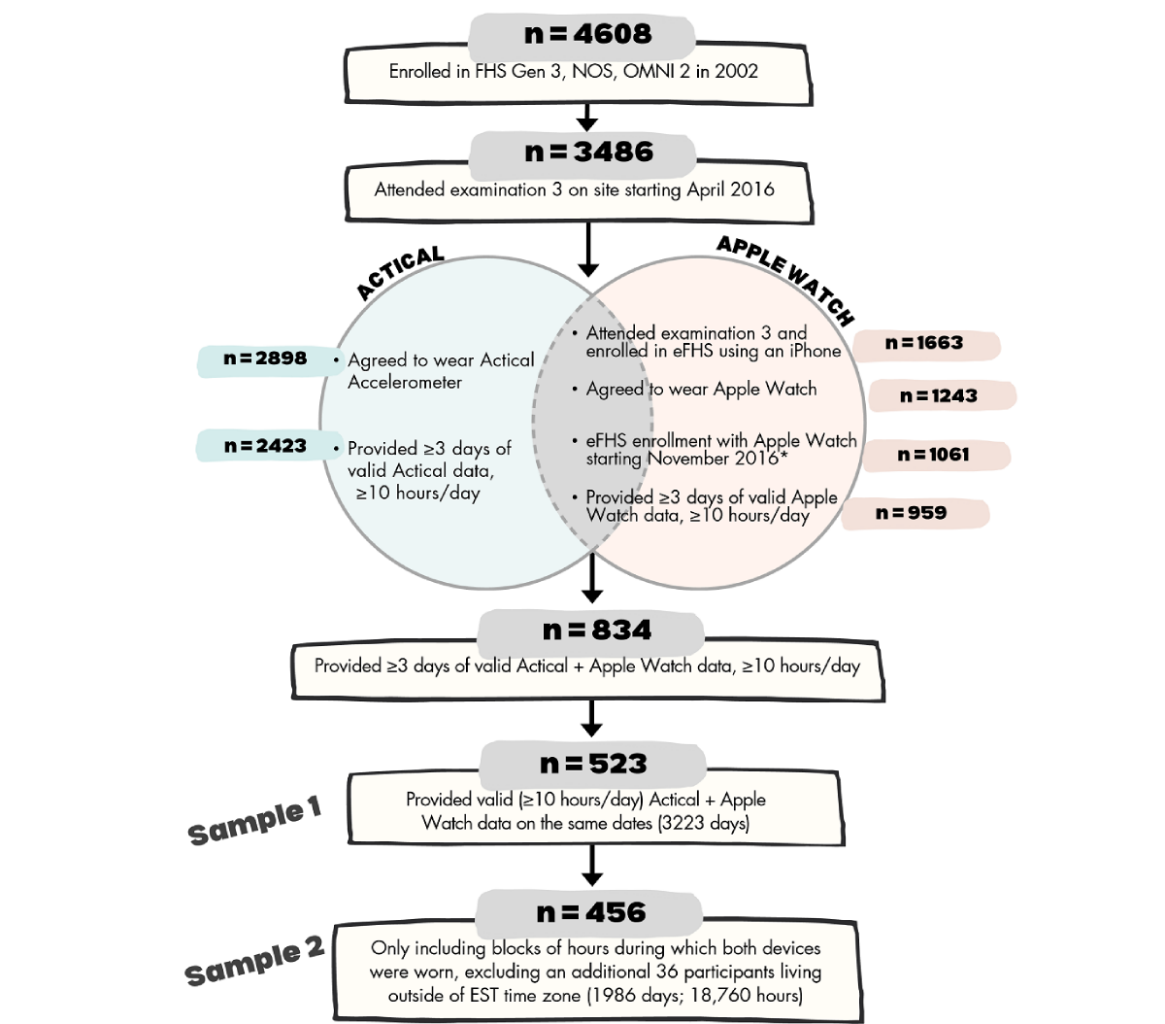
Participant flow diagram for the analysis of agreement between Apple Watch and Actical step counts. eFHS: electronic Framingham Heart Study; EST: Eastern Standard Time; FHS: Framingham Heart Study; Gen: generation; NOS: New Offspring Spouse. *Enrollment in eFHS starting in November 2016 was necessary because this was the first date Apple Watches were given out at the FHS Research Center. Participants were able to enroll in eFHS prior to this, but they were given an Apple Watch to use later (starting in November), so their Apple Watch use would not align with the Actical monitor wearing dates.

During the third in-person research examination of these cohorts (April 2016-March 2019), participants were asked to wear an Actical accelerometer for 8 consecutive days on the hip. Beginning in November 2016, as part of the electronic FHS (eFHS) ancillary study [18], participants were also asked to wear an Apple Watch (Series 0) on their wrist for up to 1 year if they owned an iPhone with a compatible iOS (version 9 or higher). Of the 3486 FHS participants examined at the Research Center for exam 3, from April 2016 to March 2019, a total of 2898 (83%) agreed to take the Actical monitor, of which n=2423 (92% of those who took the device) had “valid” steps data, meaning they wore the monitor for at least 3 days, for at least 10 hours per day (Figure 1).

In total, 1061 eFHS enrollees (since November 2016) agreed to take the Apple Watch or use their own, of which 959 (90% of those who agreed to use an Apple Watch) wore the device for at least 3 days for at least 10 hours per day during the follow-up period. A total of 834 participants had at least 3 days of “valid” data from both devices (Actical and Apple Watch). Of those, 523 participants had at least 10 hours of wear time on both devices on the same day, providing a total of 3223 person-days for our primary study sample (sample 1).

### Ethical Considerations

All participants provided written informed consent and the institutional review board at Boston University Medical Center approved the study protocols (H-32132).

### Actical Physical Activity

During the 8-day wear period, participants were asked to remove the Actical accelerometer (Philips Respironics, model numbers 198-0302-xx, Respironics Co Inc) each night for sleep and when bathing or swimming. Actical data were recorded in 30-second epochs and expressed as counts (or steps) per 30 seconds. Actical step counting has been validated against hand counting in a laboratory setting [19,20]. For sample 1, data were processed using a SAS program (SAS Institute) developed by Colley et al [21], and modified with input from collaborators [22], including nonwear time removal using the Choi algorithm [23], as explained in detail in Methods in Multimedia Appendix 1 [23]. After processing, there remained 18 hours of possible wear time per day. A valid day was defined as ≥10 hours of wear time, with ≥3 days required for inclusion in the main analysis [24].

### Apple Watch Series 0 Physical Activity

As part of the eFHS protocol, participants were asked to wear the smartwatch daily and were sent home with instructions on proper smartwatch use with advice to remove the smartwatch for charging every night. We also set up permissions for the Apple Watch app to access health information from other apps on the smartphone (ie, steps, heart rate, blood pressure, and weight) but we did not enter participant-specific data during Apple Watch setup. In contrast to data collected from the Actical, which had both counts and steps per 30-second interval, we were only able to collect Apple Watch data at the granularity of the number of steps per hour. For the Apple Watch, 1 wear hour was defined as an hour with at least 2 heart rates or at least 30 steps accumulated [25]. Unlike for Actical, there was no maximum number of wear hours chosen for the Apple Watch. A valid day was defined as ≥10 hours of wear time, with ≥3 days required for inclusion in the main analysis.

### Covariates

The covariates that were measured during the examination when Actical and Apple Watch devices were provided to participants were current smoking status, self-reported health, BMI, hypertension stage II (systolic blood pressure ≥140 mm Hg or diastolic blood pressure ≥90 mm Hg, or use of blood pressure medications) [26], diabetes mellitus (fasting plasma glucose ≥126 mg/dL and use of medications for diabetes mellitus), and prevalent cardiovascular disease. Depression status was defined as anyone with a score of 16 or greater on the Center for Epidemiological Studies Depression (CESD) scale. The physical activity index was a composite score constructed by weighing self-reported time spent in physical activity intensities over a 24-hour “typical” day [27]. Mobility limitation was defined as those self-reporting that they were unable to walk 0.5 miles without help or that they were limited a little or a lot when climbing several flights of stairs.

### Statistical Analysis

After excluding participants who did not have at least 3 days of valid data from both devices and then excluding dates on which only 1 device was worn, we were left with 523 participants (3223 person-days, sample 1). We compared the number of hours participants wore each device on average days and average steps accumulated to determine device-specific differences, reporting means and SDs or medians and IQRs.

To examine the agreement between devices on days when both devices were worn for >10 hours (sample 1), we reported the intraclass correlation (ICC) using the random-effects model in our 2 study samples and used the Lin concordance coefficient (accounting for repeated observations). We also used kappa coefficients to assess concordance between the devices in identifying participants meeting thresholds of average daily steps (at 3000, 6000, 8000, and 10,000 steps per day). Bland-Altman plots were also used to assess potential non-systematic differences between devices and provide a visual representation of these differences in steps per day and the percent differences (100 multiplied by [Apple Watch mean minus Actical steps] divided by mean steps). We assessed the limits of agreement for the Bland-Altman plot using repeated measures. Agreement of Apple Watch and Actical step counts per day was also assessed as the percent of days in which steps for each device fell within 15% agreement of one another. In personal communication with physical activity research experts (unpublished), most suggested that acceptable agreement should be set at a 5% difference level (Tudor-Locke et al [28]), with a few experts acknowledging that agreement within 15% may be considered acceptable (Breteler et al [14]). Experts polled were those who participated as an author in the meta-analysis of 15 international cohorts with accelerometer data published by Paluch et al [11]. We chose to report the more lenient agreement threshold in order to better detect variability in agreement among subsamples of our population, especially after observing the poor overall agreement within these ranges displayed in the results.

In secondary analyses, we also examined agreement between devices during hours when both devices were worn (sample 2) to account for potential differences in wear periods (by device) throughout the day. To create this sample, first, we identified blocks of time ≥3 hours each day (midnight to midnight) during which both devices were worn. We defined an hour of Actical wear as any hour with >0 step count. In this study, we defined an hour of Apple Watch wear was defined as an hour with >30 step counts or 0-30 step counts with at least 2 heart rates recorded, but there does not seem to be an established threshold used in this research field. We excluded hours for which step counts were missing (shown as NA in Figure S1 in Multimedia Appendix 1). These definitions differed because of different device-wearing locations (hip vs wrist). When devices are worn on the hip, they can show 0 step counts for prolonged periods of time when a participant is wearing the device sitting, but this is less likely to occur with a wrist-worn device. A total of 30 participants had <3 hours of overlapping wear time and were excluded (Figure 1). These 30 participants had >10 hours of Apple Watch wear time on days when the Actical was worn for >10 hours, but the Apple Watch wear hours did not have at least 3 consecutive hours. When steps were counted, each hour or 2, they were broken up by hours with heart rate measurements, but they were often missing step counts. An example of 24 hours of Actical and Apple Watch data is shown in Figure S1 in Multimedia Appendix 1. We provide further interpretation of these “interruptions” in wear time in the discussion section. Our next step was to remove the first and last hour of each ≥3-hour block because we could not determine whether they were full or partial hours. The remaining hours in that block were each used as separate data points, to provide us with steps accumulated by the 2 devices for every hour that both devices were worn. As Apple Watch (but not Actical), changes time stamps during the collection period to be consistent as people move across different time zones, we additionally excluded participants residing outside of the Eastern Standard Time Zone (n=36), which may have resulted in discordant hours being counted by each device. One extreme outlier (1 person-hour) was also removed (see Figure S2 in Multimedia Appendix 1), which did not affect results (data not shown). We repeated the analysis from sample 1.

Next, for each sample, we tested for interactions by age, sex, height, and BMI in the linear regression analysis to assess whether these factors influenced agreement between the Apple Watch and Actical device measures of total daily steps. Finally, we performed sensitivity analyses, repeating our agreement analysis in subsamples excluding participants with high or low step counts. All statistical analyses were performed with R (version 4.1.3; R Core Team), including ggplot2 (for plots), irr (for ICC), epiR (for Lin concordance correlation), and psych (for kappa coefficients) packages.

## Results

### Overview

Compared to the total FHS Gen 3, NOS, and Omni 2 cohort, participants who returned valid (ie, sufficient) data from the 2 wearable devices were on average younger, healthier (less smoking, diabetes, hypertension, cardiovascular disease, and depression), and were more likely to have completed college or received a graduate degree (Table 1). The average wear time for the Apple Watch was more than an hour longer each day than for the Actical (15.6 vs 14.4 hours; Table 1), which may be partially due to the removal of 6 hours of each 24 hours and other Actical data processing, as described in Methods in Multimedia Appendix 1.

**Table 1:** Characteristics for all FHS^a^ Gen^b^ 3 participants who attended examination 3, compared to those with valid Actical and Apple Watch data on the same date.

			FHS Gen 3 (n=3521)		FHS Gen 3 with valid Actical +Apple Watch data on the same date (sample 1, n=523)
Age (years), mean (SD)			54.5 (9.4)		51.7 (8.9)
Women, n (%)			1896 (53.9)		298 (57.0)
**Race and ethnicity, n (%)**					
	Non-Hispanic White	3233 (91.8)		478 (91.4)	
	Non-Hispanic Black	59 (1.7)		12 (2.3)	
	Hispanic or Latino	106 (3.0)		14 (2.7)	
	Asian	71 (2.0)		9 (1.7)	
	American Indian	1 (0.03)		1 (0.2)	
	Pacific Islander	2 (0.06)		0 (0)	
	More than 1 race	41 (1.2)		8 (1.5)	
	Unknown	8 (0.2)		1 (0.2)	
BMI (kg/m^2^), mean (SD)			28.6 (6.2)		28.2 (5.7)
Height (inches), mean (SD)			66.6 (3.7)		66.8 (3.6)
Mobility limitation, n (%)			703 (20)		85 (16.3)
Smoking, n (%)			234 (6.7)		27 (5.2)
**Education, n (%)**					
	Less than HS^c^	48 (1.4)		3 (0.6)	
	Completed HS	470 (13.5)		43 (8.2)	
	Some college	489 (14)		114 (21.8)	
	Bachelor’s degree	1222 (35.0)		214 (41.0)	
	Graduate or professional degree	843 (24.1)		148 (28.4)	
Married, living as married, living with partner, n (%)			2454 (70.5)		397 (76.4)
Employed full-time, n (%)			2277 (65.4)		381 (73.1)
Self-reported health (excellent), n (%)			750 (21.4)		128 (24.5)
Diabetes mellitus, n (%)			310 (8.8)		26 (5.0)
Hypertension stage II, n (%)			1095 (31.1)		112 (21.4)
Cardiovascular disease, n (%)			164 (4.7)		18 (3.4)
Depression (CESD^d^ >16), n (%)			449 (12.8)		55 (10.5)
Physical activity index (score), mean (SD)			33.9 (5.7)		33.2 (4.7)
Actical steps, median (IQR)			N/A^e^		7064 (4638-10,529)
Apple Watch steps, median (IQR)			N/A		7060 (4450-10,348)
Actical wear time (hours), mean (SD)			N/A		14.4 (1.8)
Apple Watch wear time (hours), mean (SD)			N/A		15.6 (2.6)

^a^FHS: Framingham Heart Study.

^b^Gen: generation.

^c^HS: high school.

^d^CESD: Center for Epidemiological Studies Depression.

^e^N/A: not applicable.

### Primary Analysis (Sample 1, n=523): Step Agreement Per Day of Device Wear

We observed a modest correlation (ICC 0.56, 95% CI 0.54-0.59; Table 2), but poor agreement (29.7%, n=957 of days having steps counts with ≤15% difference) between devices. Lin concordance coefficient, accounting for repeated observations, produced the same coefficients as traditional ICC for all results. The 2 devices demonstrated moderate agreement for distinguishing between participants meeting versus not meeting step per day thresholds by their average daily steps (kappa coefficient=~0.5; Table 3). The Apple Watch and Actical devices were concordant 74.8% (n=391)-84.5% (n=442) of the time, depending on the threshold (3000, 6000, 8000, and 10,000 steps per day). This reliability for distinguishing between thresholds did not change greatly if we used average daily steps (as in Table 3) or steps per person-day (as in Table S1 in Multimedia Appendix 1), but improved slightly to 77.2% (n=889) to 85.3% (n=982) if we excluded person-days in which wear time was >1 hour different between.

**Figure 2:**
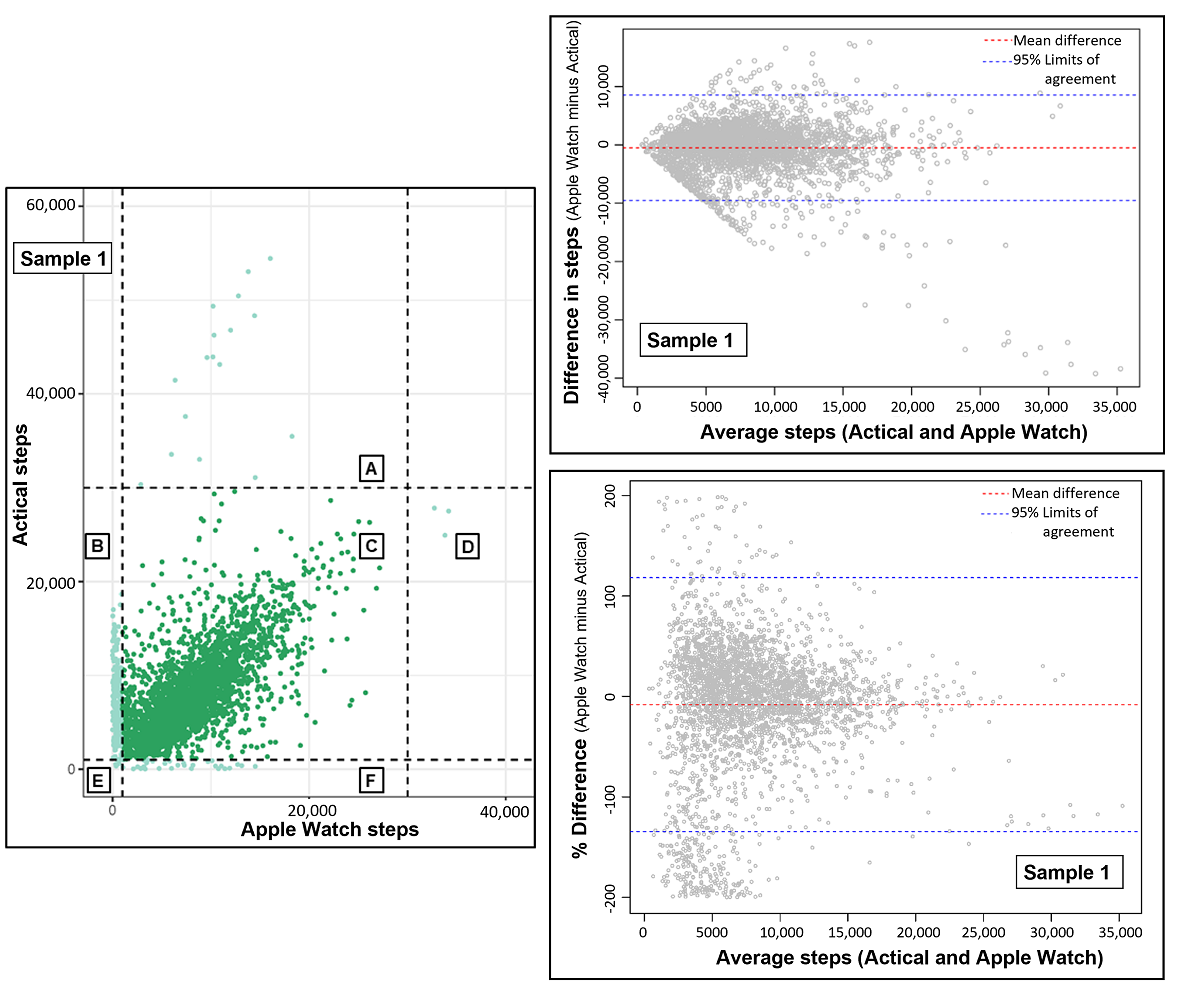
Scatterplot and Bland-Altman plots (difference and % difference) of Apple Watch steps by Actical steps accumulated on the same date (sample 1, all data, 3223 person-days, 523 participants).

On average, we observed more steps per day counted by the Actical device, with a mean difference of 499 more steps per day counter compared to the Apple Watch (Figure 2, Table 2). Limits of agreement were –9543, 8544 steps per day, meaning that differences in step counting between devices are expected to be roughly ±9000 steps in a given day of device wear. The differences in step counting between devices tended to increase with higher average steps counted, but the percent differences did not (average limits of agreement were –134.6% to 118.2% difference between step counts). There also did not appear to be a major under- or overestimation of steps by 1 device compared to the other. We observed an interaction (Table S2 in Multimedia Appendix 1; *P*<.001) between wear time and device type in their association with daily step count.

Each point represents data from 1 participant on a single date (1 person-day). In the scatterplot, dashed lines are set at 1000 and 30,000 step thresholds. Days on which participants accumulated 1000-30,000 steps are dark green and days outside that threshold are presented in light green. Sections separated by the dashed lines include the following number of person-days divided by participants: A=17/5, B=205/68, C=2963/512, D=3/2, E=4/4, and F=31/29. The Bland-Altman plots on the right show the mean difference or mean % difference (red dashed line) and the limits of agreement 95% CI (blue dashed lines). The mean % difference was calculated as 100 multiplied by (Apple Watch steps minus Actical steps) divided by (average Apple Watch and Actical steps).

**Table 2:** Agreement between steps accumulated on Actical versus Apple Watch device by participants wearing both devices on the same date^a^.

Sample and sample description			Adjusted linear regression, β (95% CI)		ICC^b^ (95% CI)		Lin concordance correlation, *r* (95% CI)		Mean difference^c^ (Bland-Altman limits of agreement)		Mean % difference^d^ (Bland-Altman limits of agreement)		Percent of Apple Watch days with a step count within 15% agreement compared to Actical, n (%)
Sample 1 (n=523 participants; n=3223 person-days): includes all days when both devices were worn for >10 hours			0.67 (0.65-0.70)		0.56 (0.54-0.59)		0.56 (0.54-0.58)		–499 (–9543 to 8544)		–8.2 (–134.6 to 118.2)		957 (29.7)
**Sample 2 (n=456 participants; n=1986 person-days; n=18,760 person-hours): only includes blocks of hours during which both devices were worn^e^**			0.97 (0.96-0.97)		0.86 (0.85-0.86)		0.86 (0.85-0.86)		20 (–844 to 884)		16.6 (–98.0 to 131.3)		5115 (27.3)
	Sample 2A (n=151 participants; n=5397 person-hours): with obesity	0.94 (0.92-0.95)		0.85 (0.84-0.86)		0.85 (0.84-0.85)		33 (–844 to 909)		18.2 (–95.5 to 131.9)		1397 (25.9)	
	Sample 2B (n=79 participants; n=2967 person-hours): with mobility limits	0.86 (0.84-0.88)		0.86 (0.83-0.88)		0.86 (0.85-0.87)		98 (–953 to 1148)		29.8 (–83.8 to 143.5)		709 (23.9)	
	Sample 2C (n=266 participants; n=11699 person-hours): without obesity or mobility limitations	0.98 (0.97-0.99)		0.85 (0.85-0.86)		0.85 (0.85-0.86)		3 (–1089 to 1096)		14.4 (–101.4 to 130.3)		3275 (28.0)	

^a^The adjusted linear regression model includes age, sex, cohort type, BMI, height, (and the difference in wear time for sample 1).

^b^ICC: intraclass correlation.

^c^Mean difference was Apple Watch steps minus Actical steps

^d^Mean % difference was 100 multiplied by (Apple Watch steps minus Actical steps) divided by (average Apple Watch and Actical steps).

^e^Sample 2: after removing hours when both devices were not being worn, we removed the first and last hours of remaining blocks of hours. We additionally excluded participants who lived outside Eastern Standard Time Zone and removed 1 data point that was an extreme outlier (Figure S1 in Multimedia Appendix 1). We used each remaining hour as a separate data point.

**Table 3:** Agreement of Actical and Apple Watch devices to identify participants meeting average daily step thresholds (sample 1, n=523 participants; 3223 person-days).

Step per day threshold	Percent concordance for “meets the PA^a^ threshold” as measured by the 2 devices, n (%)	Kappa coefficients (95% CI) for “meets the PA threshold” as measured by the 2 devices
3000 steps per day	442 (84.5)	0.12 (0.01-0.22)
6000 steps per day	396 (75.7)	0.46 (0.38-0.54)
8000 steps per day	391 (74.8)	0.49 (0.41-0.56)
10,000 steps per day	426 (81.5)	0.49 (0.40-0.58)

^a^PA: physical activity.

### Secondary Analysis (Sample 2, n=456): Step Agreement Per Hour of Device Wear

**Figure 3:**
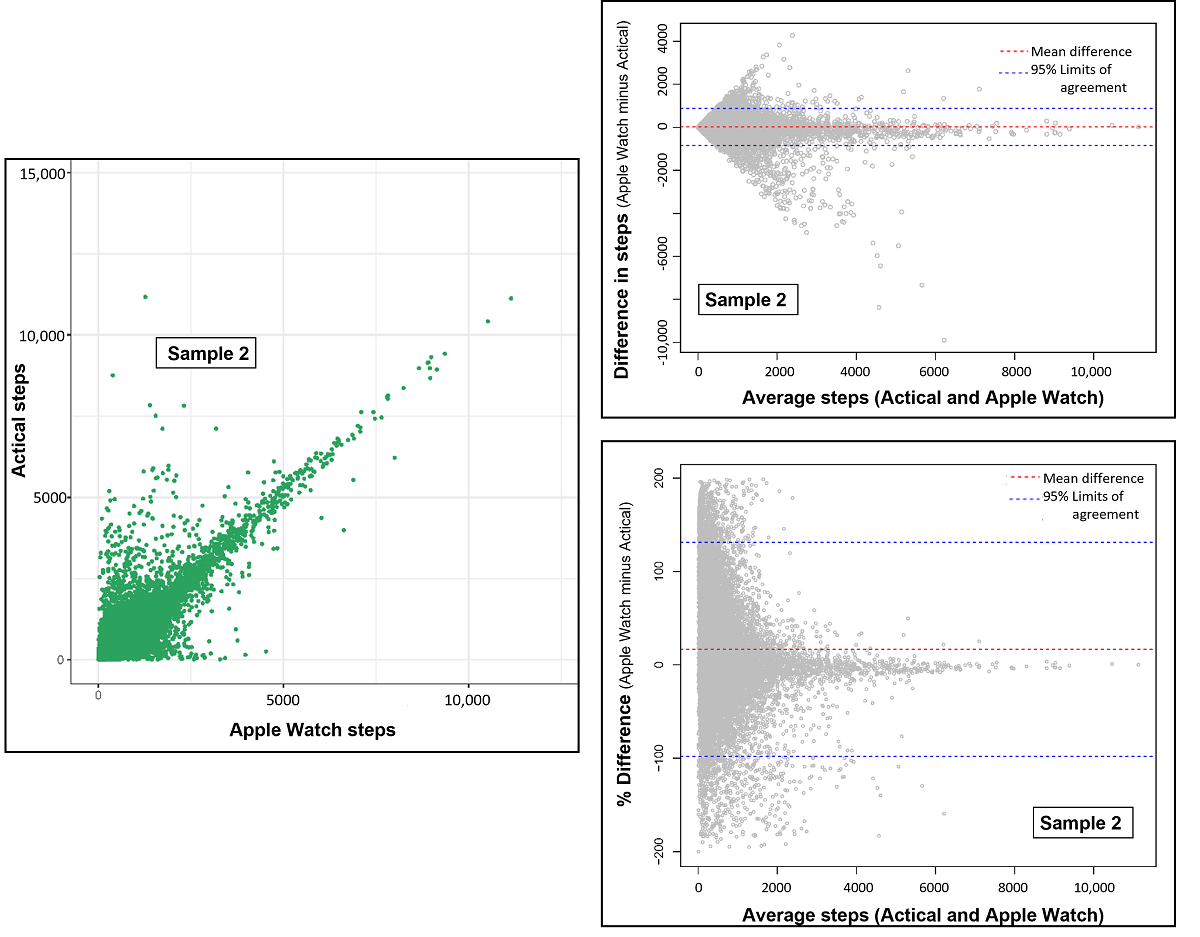
Scatterplot and Bland-Altman plot (difference and % difference) of Apple Watch steps by Actical steps accumulated during hours when both devices were worn (sample 2, n=456 participants; n=1986 person-days; n=18,760 person-hours).

We conducted secondary analyses to explore the agreement between devices, with differences in wear time minimized. We assessed agreement between devices for each hour during which both devices were worn (456 participants, 1986 person-days, 18,760 person-hours, Table 2 and Figure 3). Among hours when both devices were worn, the correlation of absolute step counts between devices was much stronger (ICC 0.86, 95% CI 0.85-0.86, Table 2) than it was for sample 1, but the agreement of steps counted per hour was still poor (only 27.3%, n=5115 of hours having step counts with ≤15% difference) between devices. The mean difference in step count between devices was only 20 steps per hour, but limits of agreement were large (–844, 884 steps per hour) and a 16.6% difference (–98, 131.3% limits of agreement) between Apple Watch and Actical step counting on hours when both devices were worn.

Each point represents data from a single hour (1 person-hour). The Bland-Altman plots on the right show the mean difference or mean % difference (red dashed line) and the limits of agreement 95% CI (blue dashed lines). The mean % difference was calculated as 100 multiplied by (Apple Watch steps minus Actical steps) divided by (average Apple Watch and Actical steps).

Next, we assessed potential interactions (in sample 2, Table S2 in Multimedia Appendix 1), observing interactions by obesity status and mobility status (*P*<.001). We observed that correlations were similar regardless of these factors (samples 2A-2C, Table 2), but the agreement of step counts with ≤15% difference between devices was slightly worse for participants with obesity (n=1397, 25.9% agreement) or self-reported mobility limitations (n=709, 23.9% agreement), compared to those with neither (n=3275, 28.0% agreement).

### Sensitivity Analyses: Exploration of Days With Low Step Counts and Large Differences in Step Count

Despite a strong correlation in step counts, there was substantial variability between devices in terms of device agreement, as demonstrated in the total sample 1, Figure 2. We observed 17 person-days with >30,000 steps per day by Actical but <20,000 steps per day by Apple Watch, representing days from 5 participants (section A in Figure 2, Figure S3 and Table S3 in Multimedia Appendix 1). In sensitivity analyses excluding data from these 5 participants, the ICC improved slightly for samples 1 and 2, but the percent of days or hours during which the devices agreed within 15% only improved by <1% (Table S4 in Multimedia Appendix 1). Very few days (~10%) met the stricter 5% threshold for agreement between devices and agreement was further reduced when only observing days when the Apple Watch was worn 5-10 hours and Actical was worn >10 hours.

Other substantial variability we observed between device counting by Actical compared to Apple Watch was observed in a large number of days during which 1 device counted <1000 steps and the other device counted >1000 steps (sections B and F in Figure 2). In Figures S4 and S5 in Multimedia Appendix 1, we show scatterplots for hours when both devices were worn. During most hours represented in sections B and F in Figure 2, the devices were either being worn at different times of the day or there were interruptions in step counting. Furthermore, when observing hours from “other days” of those participants who had days that fell into sections B or F (Figures S4 and S5 in Multimedia Appendix 1), the pattern appears similar to the scatterplot for the overall sample 2 (Figure 3). Exclusion of participants who contributed days that fell into sections A, B, D, E, or F in Figure 2 (those with step counts <1000 or >30,000 by either device) did not improve agreement results greatly either, improving the percent of days on which the devices agreed within 15% only up to 32.2% of all days (Table S4 in Multimedia Appendix 1).

## Discussion

### Principal Findings

Consumer accelerometer devices are being used by millions of people to track their physical activity levels and progress toward public health recommendations or personal goals. These devices have been validated in laboratory settings against research-grade devices, but few studies have explored how consumer and research-grade accelerometer step counting compares when participants are living out in the community.

In this study, we observed poor overall agreement between steps counted by Actical and Apple Watch (Series 0) devices. Larger between-device differences were seen when the step count was higher, but the percent difference did not increase. However, our results suggest that we can expect the 2 devices to classify individuals into the same step thresholds about 75%-85% of the time. Results such as these may be important to consider when translating future step guidelines to the public using consumer brand devices. The limitations in agreement among accelerometer devices may be less important when they are used by individuals to determine the achievement of a recommended number of steps or for the purposes of tracking their step count over time.

The agreement we observed in this study in a free-living environment was worse than previous laboratory-based studies of consumer-grade devices comparing them to hand-counted steps or research accelerometer devices [13,15]. However, 1 study observed that even when testing the consistency of step counting in the same device, wearing the device at different locations (wrist vs hip) can result in inconsistencies in device step counting [29]. The difference in device location alone may have contributed greatly to the poor agreement of step counting between devices in our study.

Similar to our design, 1 study by Breteler et al [14], examined Apple Watch step counting in a free-living setting (wrist-worn) in comparison to other accelerometers worn on the hip. In this study, 30 healthy participants (mean age 40 years) wearing multiple devices over a 3-day period observed a median absolute relative difference of 7.7% comparing the Apple Watch to the ActiGraph (similar to our mean relative difference results comparing Apple Watch to Actical). However, they did not report the limits of agreement for this relative difference. Other devices they tested had a median absolute relative difference >15% [14]. A low mean or median relative difference indicates low bias (lack of systematic over- or undercounting by 1 device), but only limits of agreement can inform about the precision of agreement. Breteler et al [14] reported the mean difference in step counting was 968 more steps per day counted by the Apple Watch with limits of agreement ±6000 steps per day (compared to ActiGraph), which is almost as high as the limits of the agreement we observed in our study sample 1 (compared to Actical). Investigators in that study observed that Apple Watch devices added steps overnight when other devices were not counting any steps, which could have been due to the delayed transmission of step count data. We did not observe the same phenomenon in our data, which may be due to us using an older Apple Watch device model. In our study, we observed that some participants had long periods of consecutive hours with heart rate data, but 0 step counts (meaning the Apple Watch device was being worn) and had long periods of consecutive Actical step data >0 during this time. We suspect that the Apple Watch step data were either not being recorded or transmitted during these time periods or were delayed by many hours. In order for Apple Watch Series 0 data to be recorded or transmitted, the participant’s smartphone needed to be charged, connected to the internet, and unlocked. This finding has important implications for future research teams when analyzing data from other mobile health devices.

Although we observed poor overall agreement (due to wide limits of agreement) in our study and in Breteler et al [14] we also reported low bias due to low mean difference and percent difference. However, individual differences in gait, which may be in part due to older age, mobility limitations, or body stature (influenced by sex, height, and body composition), might introduce systematic bias into the measurement of steps in the community and should be considered in future studies [30-32]. Accelerometers have different sensitivities to slow gait speeds or low-frequency movement [29], even when tested in a laboratory in which gait differences are minimized. We observed slightly worse agreement between devices for individuals who were obese or self-reported mobility limitations. An individual’s usual cadence and the amount of time they spend participating in movement activities other than ambulation (such as household chores or other multidirectional activities) may also influence step detection in certain accelerometer devices [33-35]. Although individuals with mobility limitations and other conditions that alter gait (eg, obesity) only worsened agreement slightly, the overall influence of gait on how these devices count steps may partially explain the poor agreement between devices.

Agreement of step-counting devices has implications for future updates of the Physical Activity Guidelines. Advancements in technology and the widespread availability of consumer wearable devices make physical activity monitoring feasible in research or clinical settings and for individuals in the community. During the development of the 2018 Physical Activity Guidelines, it was determined that there was insufficient evidence to create a guideline for health promotion based on step count [1]. However, an estimation by Tudor-Locke et al [36] suggests that the MVPA guidelines can be met by adults who walked a minimum of ~7000-8000 steps per day. Furthermore, a 2022 meta-analysis of 15 observational cohort studies (including FHS) using research-grade physical activity monitors (eg, ActiGraph, Actical, and Activ-PAL), reported that individuals achieving ≥8000 (vs <8000) steps per day in middle age had the lowest risk of mortality [11]. In older adults, a lower threshold of ≥6000 steps per day was associated with almost 50% lower risk of death compared to older adults who walked less. The study, which was the largest meta-analysis of its kind, may serve as evidence to support future guidelines as to the number of steps adults should walk each day.

Although we now have some evidence that achieving step thresholds from 6000 to 8000 steps per day is associated with lower mortality [11], creating step guidelines is complicated by the observation that individuals in the community do not use the same research-grade devices as used in many prior studies. Instead, the public uses popular consumer activity trackers, such as Fitbit (Google), Apple Watch, and Garmin among other devices. Although these consumer devices have been well validated for the measurement of steps in laboratory settings [13-15], it has not been clear whether the steps counted by these consumer devices are comparable to steps counted by research-grade devices in free-living settings [37]. Unfortunately, it does not appear to be a simple fix to “convert” steps measured by a research device to those measured by a consumer device, based on the wide limits of agreement. Despite the poor overall agreement of step counting between devices, favorably, the devices had a substantially better agreement in identifying who meets thresholds between 6000 and 10,000 steps per day, with an agreement for ~75%-82% of individuals. These thresholds may serve as targets for future public health recommendations.

### Strengths and Limitations

Strengths of our investigation include the large sample size and the study being conducted in a community setting, which increases the generalizability of the findings. However, the homogeneous nature of our study cohort, who were mostly from 1 geographic location, were generally healthier and more highly educated than the general US population and were mostly of European ancestry, may limit generalizability to more diverse populations. Another strength was our use of different person-day samples to examine different questions such as comparing step counts between devices when worn for a comparable number of hours and observing the influence of different wearing behaviors on step count agreement. The lack of control of wear time and differences in wear time observed between the devices can be viewed as both a weakness (because wear time affects step accumulation) and strength (in that it demonstrates the differences that may be inherent in real-world device use). Similarly, as noted earlier, another difference between these devices was their placement on the wrist versus the hip, which may have also contributed to the variation. However, the device placement locations add another real-world element to our study design.

Wear time per day was longer, on average, for the Apple Watch, which may, in part, be due to wearing during sleeping hours. In our analysis, sleeping hours were removed from the Actical total wear time, but not from the Apple Watch. We asked participants to remove the Actical device when they bathed, swam, or slept. These instructions were not given to participants for the Apple Watch, although participants may have chosen to do so. The Apple Watch is waterproof, but the battery does not typically last much longer than 24 hours, so most participants likely took off the Apple Watch to charge at night. If the Apple Watch battery was not charged, a participant might not have worn the device and may have missed opportunities to record steps walked. The Actical battery did not need to be charged during the week that participants wore the device, which may have affected when it was worn compared to the Apple Watch. On the other hand, the Actical device was worn on a belt around the waist or hip, compared to the Apple Watch, worn on the wrist, either of which can be cumbersome, causing some participants to remove the device or wear it improperly (eg, loosely). It is unclear which placement site is preferred by the research community [38]. Although we sent participants home with instructions for when to take on and off the devices and the location where they should be worn, we did not emphasize that they should ensure a snug fit. Another possibility is that there could be calibration issues with some of the devices (Actical and Apple Watch could have drifted from factory calibration). A comparison of agreement results between samples 1 and 2 makes it clear that it is unlikely that the poor agreement was explained by participants wearing the devices during different times of day or activities. But it is also evident that some of the differences in step counting by these devices may have been due to Bluetooth connectivity errors in the recording or transmission of step data, which led to very low steps counted by the Apple Watch.

Features of physical activity monitors are also important considerations. The Apple Watch device used in this investigation has many applications, including allowing participants to see step counts as they were accumulated (there was no visual display on the Actical device) and other functionalities. The availability of these features may also influence when the device is worn and how many steps are taken. We did observe that of the participants who agreed to either device, a roughly equal proportion of participants (~90%) wore those devices for ≥3 days for at least 10 hours per day. However, studies have shown that features such as a display showing step progress and encouragement (ie, nudges) to stand or move may increase both wearing and stepping behavior, especially over the short term, which may influence results from studies using consumer devices that tend to have these features.

Our study provided us with many lessons that we hope to communicate with investigators using accelerometers. An unexpected finding was that the agreement between these physical activity monitors only improved slightly after we limited differences in wear time between the devices. When experts develop public health guidelines for the number of steps to walk each day, they must consider that devices do not all record steps equivalently and that the type of device, wearing location, or mode (ie, watch, belt, or smartphone app), battery life, Bluetooth connectivity issues, other features of the device, and gait differences of participants may all influence when the device is worn and how many steps are counted. Moreover, we did not enter participant-specific data (ie, height, weight, age, and sex) when setting up the Apple Watch or Actical devices. However, the Apple Watch may have accessed this type of data from other health-related apps on a participant’s smartphone. It is also important to note that we studied older versions of the devices, both of which are no longer supported by their manufacturers. Hopefully, newer device models may have overcome some of the limitations of the accelerometers we studied; we used Apple Watch Series 0 during data collection, but they have already transitioned to Series 8. In future research, it may also be important to emphasize proper wear of devices and input relevant participant-specific information during device setup for improved precision.

### Conclusions

Our investigation suggests that overall agreement between steps counted by the Actical and Apple Watch Series 0 devices was poor, but agreement between devices was much stronger for distinguishing who meets certain step thresholds. Many large cohort studies have used the Actical device and other research and consumer devices to observe thresholds of physical activity (steps per day) that are associated with health outcomes [11,39-41]. Lessons learned from our investigation should be considered when translating thresholds of steps counted using the Actical to guidelines for members of the community using consumer devices, including the Apple Watch. Future studies should explore the agreement among other devices in the community setting and explore the role of interruptions in connectivity, calibration, and factors affecting gait, such as age, sex, frailty or mobility status, BMI, and height on the accuracy of step count and agreement among devices. However, another important future direction should be the increased use of consumer accelerometer devices in research in order to replicate recent meta-analyses reporting the higher risk of mortality among physically inactive individuals (measured using research-grade devices) [11,42]. Studies such as All of Us and the Risk Underlying Rural Areas Longitudinal (RURAL) Heart and Lung Study that use Fitbit devices, for example, will be extremely useful in the development and translation of future physical activity step guidelines [41].

The good news is that the impact of these challenges in measuring steps may be minimized when accelerometers are used by individuals for the purposes of tracking the changes in their physical activity over time, which eliminates the impact of gait differences (unless gait changes), factory calibration issues (if the same device is used), and presumably connectivity issues would remain consistent, limiting their impact too.

## Appendix

Multimedia Appendix 1 Additional methods and results.

## Abbreviations

CESD: Center for Epidemiological Studies Depression
eFHS: electronic Framingham Heart Study
FHS: Framingham Heart Study
Gen: generation
ICC: intraclass correlation
MVPA: moderate to vigorous physical activity
NOS: New Offspring Spouses
RURAL: Risk Underlying Rural Areas Longitudinal

## Data Availability

The data sets generated and analyzed during this study are available from the corresponding author on reasonable request. The Framingham Heart Study makes data available through the online repositories BioLINCC and dbGap.

## References

[1] Physical Activity Guidelines Advisory Committee (2018). 2018 Physical Activity Guidelines Advisory Committee Scientific Report.

[2] Tucker JM, Welk GJ, Beyler NK (2011). Physical activity in U.S.: adults compliance with the physical activity guidelines for Americans. Am J Prev Med.

[3] Zenko Z, Willis EA, White DA (2019). Proportion of adults meeting the 2018 physical activity guidelines for Americans according to accelerometers. Front Public Health.

[4] Lorbergs AL, Prorok JC, Holroyd-Leduc J, Bouchard DR, Giguere A, Gramlich L, Keller H, Tang A, Racey M, Ali MU, Fitzpatrick-Lewis D, Sherifali D, Kim P, Muscedere J (2022). Nutrition and physical activity clinical practice guidelines for older adults living with frailty. J Frailty Aging.

[5] Fanning J, Nicklas BJ, Rejeski WJ (2022). Intervening on physical activity and sedentary behavior in older adults. Exp Gerontol.

[6] Office of the Surgeon G (2015). The Surgeon General's Call to Action to Promote Walking and Walkable Communities.

[7] Fox S, Duggan M (2013). Tracking for health. Pew Research Center.

[8] Abril EP (2016). Tracking myself: assessing the contribution of mobile technologies for self-trackers of weight, diet, or exercise. J Health Commun.

[9] (2022). Connected consumer survey 2023. Deloitte Insights.

[10] Bassett DR, Toth LP, LaMunion SR, Crouter SE (2017). Step counting: a review of measurement considerations and health-related applications. Sports Med.

[11] Paluch AE, Bajpai S, Bassett DR, Carnethon MR, Ekelund U, Evenson KR, Galuska DA, Jefferis BJ, Kraus WE, Lee IM, Matthews CE, Omura JD, Patel AV, Pieper CF, Rees-Punia E, Dallmeier D, Klenk J, Whincup PH, Dooley EE, Pettee Gabriel K, Palta P, Pompeii LA, Chernofsky A, Larson MG, Vasan RS, Spartano N, Ballin M, Nordström P, Nordström A, Anderssen SA, Hansen BH, Cochrane JA, Dwyer T, Wang J, Ferrucci L, Liu F, Schrack J, Urbanek J, Saint-Maurice PF, Yamamoto N, Yoshitake Y, Newton RL, Yang S, Shiroma EJ, Fulton JE, Steps for Health Collaborative (2022). Daily steps and all-cause mortality: a meta-analysis of 15 international cohorts. Lancet Public Health.

[12] Hall KS, Hyde ET, Bassett DR, Carlson SA, Carnethon MR, Ekelund U, Evenson KR, Galuska DA, Kraus WE, Lee IM, Matthews CE, Omura JD, Paluch AE, Thomas WI, Fulton JE (2020). Systematic review of the prospective association of daily step counts with risk of mortality, cardiovascular disease, and dysglycemia. Int J Behav Nutr Phys Act.

[13] El-Amrawy F, Nounou MI (2015). Are currently available wearable devices for activity tracking and heart rate monitoring accurate, precise, and medically beneficial?. Healthc Inform Res.

[14] Breteler MJ, Janssen JH, Spiering W, Kalkman CJ, van Solinge WW, Dohmen DA (2019). Measuring free-living physical activity with three commercially available activity monitors for telemonitoring purposes: validation study. JMIR Form Res.

[15] Xie J, Wen D, Liang L, Jia Y, Gao L, Lei J (2018). Evaluating the validity of current mainstream wearable devices in fitness tracking under various physical activities: comparative study. JMIR Mhealth Uhealth.

[16] Splansky GL, Corey D, Yang Q, Atwood LD, Cupples LA, Benjamin EJ, D'Agostino RS, Fox CS, Larson MG, Murabito JM, O'Donnell CJ, Vasan RS, Wolf PA, Levy D (2007). The third generation cohort of the National Heart, Lung, and Blood Institute's Framingham Heart Study: design, recruitment, and initial examination. Am J Epidemiol.

[17] Dawber TR, Kannel WB (1966). The Framingham study an epidemiological approach to coronary heart disease. Circulation.

[18] McManus DD, Trinquart L, Benjamin EJ, Manders ES, Fusco K, Jung LS, Spartano NL, Kheterpal V, Nowak C, Sardana M, Murabito JM (2019). Design and preliminary findings from a new electronic cohort embedded in the Framingham Heart Study. J Med Internet Res.

[19] Esliger DW, Probert A, Connor Gorber S, Bryan S, Laviolette M, Tremblay MS (2007). Validity of the actical accelerometer step-count function. Med Sci Sports Exerc.

[20] Johnson M, Meltz K, Hart K, Schmudlach M, Clarkson L, Borman K (2015). Validity of the actical activity monitor for assessing steps and energy expenditure during walking. J Sports Sci.

[21] Colley RC, Tremblay MS (2011). Moderate and vigorous physical activity intensity cut-points for the actical accelerometer. J Sports Sci.

[22] Evenson KR, Sotres-Alvarez D, Deng YU, Marshall SJ, Isasi CR, Esliger DW, Davis S (2015). Accelerometer adherence and performance in a cohort study of US Hispanic adults. Med Sci Sports Exerc.

[23] Choi L, Liu Z, Matthews CE, Buchowski MS (2011). Validation of accelerometer wear and nonwear time classification algorithm. Med Sci Sports Exerc.

[24] Hart TL, Swartz AM, Cashin SE, Strath SJ (2011). How many days of monitoring predict physical activity and sedentary behaviour in older adults?. Int J Behav Nutr Phys Act.

[25] Lin H, Sardana M, Zhang Y, Liu C, Trinquart L, Benjamin EJ, Manders ES, Fusco K, Kornej J, Hammond MM, Spartano NL, Pathiravasan CH, Kheterpal V, Nowak C, Borrelli B, Murabito JM, McManus DD (2020). Association of habitual physical activity with cardiovascular disease risk. Circ Res.

[26] Whelton PK, Carey R, Aronow W, Casey DJ, Collins KJ, Dennison Himmelfarb C, DePalma SM, Gidding S, Jamerson KA, Jones DW, MacLaughlin EJ, Muntner P, Ovbiagele B, Smith SJ, Spencer CC, Stafford RS, Taler SJ, Thomas RJ, Williams KJ, Williamson JD, Wright JT (2018). 2017 ACC/AHA/AAPA/ABC/ACPM/AGS/APhA/ASH/ASPC/NMA/PCNA guideline for the prevention, detection, evaluation, and management of high blood pressure in adults: executive summary: a report of the American College of Cardiology/American Heart Association Task Force on clinical practice guidelines. Hypertension.

[27] Kannel WB, Belanger A, D'Agostino R, Israel I (1986). Physical activity and physical demand on the job and risk of cardiovascular disease and death: the Framingham study. Am Heart J.

[28] Tudor-Locke C, Sisson SB, Lee SM, Craig CL, Plotnikoff RC, Bauman A (2006). Evaluation of quality of commercial pedometers. Can J Public Health.

[29] Mora-Gonzalez J, Gould ZR, Moore CC, Aguiar EJ, Ducharme SW, Schuna JJ, Barreira TV, Staudenmayer J, McAvoy CR, Boikova M, Miller TA, Tudor-Locke C (2022). A catalog of validity indices for step counting wearable technologies during treadmill walking: the CADENCE-adults study. Int J Behav Nutr Phys Act.

[30] Tyo BM, Fitzhugh EC, Bassett DJ, John D, Feito Y, Thompson DL (2011). Effects of body mass index and step rate on pedometer error in a free-living environment. Med Sci Sports Exerc.

[31] Pomeroy J, Brage S, Curtis JM, Swan PD, Knowler WC, Franks PW (2011). Between-monitor differences in step counts are related to body size: implications for objective physical activity measurement. PLoS One.

[32] Feito Y, Bassett DR, Thompson DL, Tyo BM (2012). Effects of body mass index on step count accuracy of physical activity monitors. J Phys Act Health.

[33] Hickey A, John D, Sasaki JE, Mavilia M, Freedson P (2016). Validity of activity monitor step detection is related to movement patterns. J Phys Act Health.

[34] Dall PM, McCrorie PRW, Granat MH, Stansfield BW (2013). Step accumulation per minute epoch is not the same as cadence for free-living adults. Med Sci Sports Exerc.

[35] Fokkema T, Kooiman TJM, Krijnen WP, VAN DER Schans CP, DE Groot M (2017). Reliability and validity of ten consumer activity trackers depend on walking speed. Med Sci Sports Exerc.

[36] Tudor-Locke C, Craig CL, Brown WJ, Clemes SA, De Cocker K, Giles-Corti B, Hatano Y, Inoue S, Matsudo SM, Mutrie N, Oppert J, Rowe DA, Schmidt MD, Schofield GM, Spence JC, Teixeira PJ, Tully MA, Blair SN (2011). How many steps/day are enough? For adults. Int J Behav Nutr Phys Act.

[37] Middelweerd A, VAN DER Ploeg HP, VAN Halteren A, Twisk JWR, Brug J, Te Velde SJ (2017). A validation study of the fitbit one in daily life using different time intervals. Med Sci Sports Exerc.

[38] Schrack JA, Cooper R, Koster A, Shiroma EJ, Murabito JM, Rejeski WJ, Ferrucci L, Harris TB (2016). Assessing daily physical activity in older adults: unraveling the complexity of monitors, measures, and methods. J Gerontol A Biol Sci Med Sci.

[39] Cuthbertson CC, Moore CC, Sotres-Alvarez D, Heiss G, Isasi CR, Mossavar-Rahmani Y, Carlson JA, Gallo LC, Llabre MM, Garcia-Bedoya O, Farelo DG, Evenson KR (2022). Associations of steps per day and step intensity with the risk of diabetes: the hispanic community health study / study of latinos (HCHS/SOL). Int J Behav Nutr Phys Act.

[40] Spartano NL, Demissie S, Himali JJ, Dukes KA, Murabito JM, Vasan RS, Beiser AS, Seshadri S (2019). Accelerometer-determined physical activity and cognitive function in middle-aged and older adults from two generations of the Framingham heart study. Alzheimers Dement (N Y).

[41] Master H, Annis J, Huang S, Beckman JA, Ratsimbazafy F, Marginean K, Carroll R, Natarajan K, Harrell FE, Roden DM, Harris P, Brittain EL (2022). Association of step counts over time with the risk of chronic disease in the all of US research program. Nat Med.

[42] Ekelund U, Tarp J, Steene-Johannessen J, Hansen BH, Jefferis B, Fagerland MW, Whincup P, Diaz KM, Hooker SP, Chernofsky A, Larson MG, Spartano N, Vasan RS, Dohrn IM, Hagströmer M, Edwardson C, Yates T, Shiroma E, Anderssen SA, Lee IM (2019). Dose-response associations between accelerometry measured physical activity and sedentary time and all cause mortality: systematic review and harmonised meta-analysis. BMJ.

