# Supplementary material for: Agreement Between Apple Watch and Actical Step Counts in a Community Setting: Cross-Sectional Investigation From the Framingham Heart Study
Source: JMIR Biomed Eng. 2024 Jul 24;9:e54631. doi: 10.2196/54631 (PMC11306942; doi:10.2196/54631)
Supplement: Multimedia Appendix 1 [file biomedeng_v9i1e54631_app1.docx]

**Supplemental Materials for the manuscript:**

**Agreement between Apple Watch and Actical step counts in a community setting: The Framingham Heart Study**

Nicole L. Spartano PhD,^1,2^ Yuankai Zhang MA,^3^ Chunyu Liu PhD,^3^ Ariel Chernofsky PhD,^3^ Honghuang Lin PhD,^4^ Ludovic Trinquart PhD MPH,^5,6^ Belinda Borrelli PhD,^7^ Chathurangi H. Pathiravasan PhD,^3^ Vik Kheterpal, MD,^8^ Christopher Nowak PhD,^8^ Ramachandran S Vasan MD,^1,10^ Emelia J. Benjamin MD ScM,^1,9^ David D. McManus MD ScM,^4,11^ Joanne M. Murabito MD ScM^1,12^

**Table of Contents:**

**Supplemental Methods**

**Supplemental Table 1.** Agreement between the Apple Watch and Actical devices in identifying person-days on which 3000, 6000, 8000, or 10000 step/day thresholds were met (Sample 1, n=3223 person-days, 523 participants)

**Supplemental Table 2.** Interactions by factors in adjusted linear regression of the association of steps accumulated by Actical with steps accumulated by Apple Watch by devices worn on the same day (Sample 1) or same hour (Sample 2)

**Supplemental Table 3.** Characteristics for study samples assessed in this study, including detailed height information

**Supplemental Table 4.** Agreement between steps accumulated on Actical vs. Apple Watch device by participants wearing both devices on the same date (Samples 1 and 2 with additional exclusions)

**Supplemental Figure 1.** Example of 24 hours of Actical and Apple Watch data from a participant who was included in Sample 1, but not in Sample 2.

**Supplemental Figure 2:** The scatteplot shown in Figure 3, but including the single hour that was removed as an outlier (circled in red)

**Supplemental Figure 3.** Scatterplot of Apple Watch steps by Actical steps accumulated during hours when both devices were worn, only for participants who fell into section A in Figure 2

**Supplemental Figure 4:** Scatterplot of Apple Watch steps by Actical steps accumulated during hours when both devices were worn, only for participants who fell into sections B in Figure 2 (days when Apple Watch counted <1000 steps and Actical counted >1000 steps)

**Supplemental Figure 5:** Scatterplot of Apple Watch steps by Actical steps accumulated during hours when both devices were worn, only for participants who fell into sections F in Figure 2 (days when Actical counted <1000 steps and the Apple Watch counted >1000 steps)

**Supplemental Methods**

**Actical physical activity:**

To process Actical data for analyses involving Sample 1, non-wear time was removed from data processing if it met criteria defined according to an algorithm created by Choi et al.,(23) which removed periods of consecutive zero counts lasting for at least 90 minutes, allowing for short time intervals with nonzero counts lasting up to 2 minutes if no counts were detected during both 30 minutes before and after the nonzero intervals, all within those 90 minutes. For example, a non-wear window could be longer than 90 minutes, as long as the first 30 minutes and last 30 minutes had no nonzero counts. After applying the Choi algorithm, we created a sliding window of 6 hours each 24 hours (starting at 10 pm on the first day and each subsequent day), during which the lowest total number of counts were accumulated. That 6-hour window was removed completely as non-wear time for each day. These windows included time already defined as “non-wear time” by the Choi algorithm. After processing, there remained 18 hours of possible wear time per day. For analyses involving Sample 2, the processing to remove potential non-wear time, as described above, was not conducted.

**Apple Watch physical activity:**

As mentioned in the methods, participants were asked to wear the Apple Watch device for one year. Of the 1243 participants who agreed to wear an Apple Watch, 944 participants wore the device for at least 5 hours on at least 30 days out of the year, and 913 of these participants actually wore the device for ≥10 hours on ≥30 days. Using more strict criteria, a total of 363 participants wore the device for ≥10 hours on ≥5 days per month for the entire year (all 12 months).

**References**

23. Choi L, Liu Z, Matthews CE, Buchowski MS. Validation of accelerometer wear and nonwear time classification algorithm. Medicine and science in sports and exercise. 2011;43(2):357-64.

**Supplemental Table 1.** Agreement between the Apple Watch and Actical devices in identifying person-days on which 3000, 6000,8000, or 10000 step/day thresholds were met (Sample 1, n=3223 person-days, 523 participants)

| **Threshold to meet the PA guidelines** | **% Concordance for**  **“meets the PA guidelines”**  **between the two devices** | **Kappa coefficients (95% CI)**  **For “meeting the PA guidelines”**  **as measured by the two devices** |
| --- | --- | --- |
| **3000 steps/day**  **Sample 1**  **Sample 1*** | 2647 (82.1%)  982 (85.3%) | 0.19 (0.15, 0.24)  0.22 (0.14, 0.30) |
| **6000 steps/day**  **Sample 1**  **Sample 1*** | 2402 (74.5%)  889 (77.2%) | 0.47 (0.44, 0.50)  0.51 (0.46, 0.56) |
| **8000 steps/day**  **Sample 1**  **Sample 1*** | 2458 (76.3%)  889 (77.2%) | 0.51 (0.48, 0.54)  0.54 (0.49, 0.59) |
| **10000 steps/day**  **Sample 1**  **Sample 1*** | 2617 (81.2%)  947 (82.3%) | 0.53 (0.50, 0.57)  0.58 (0.53, 0.64) |

*Sample 1, excluding days with ≥1-hour difference in wear time, n=1151 person-days, 451 participants

**Supplemental Table 2.** Interactions by factors in adjusted linear regression of the association of steps accumulated by Actical with steps accumulated by Apple Watch by devices worn on the same day (Sample 1) or same hour (Sample 2)

| **Interaction factors** | **Sample 1**  **N=3223 person-days, N=523 participants**  **(p-value)** | **Sample 2**  **N=18760 person-hours, 1986 person-days,**  **N=456 participants**  **(p-value)** |
| --- | --- | --- |
| Age, years (continuous) | 0.000 (0.77) | 0.000 (0.83) |
| Sex (men vs. women) | -0.013 (0.66) | -0.012 (0.08) |
| Height, cm (continuous) | 0.001 (0.74) | -0.001(0.15) |
| BMI, kg/m^2^ (<30 vs. ≥30) | -0.010 (0.79) | -0.034 (0.0001) |
| Mobility limitation, % | 0.047 (0.25) | 0.121 (<0.0001) |
| Difference in wear time between devices, h | -0.029 (<0.0001) | ~~--~~ |

**Supplemental Table 3.** Characteristics for study samples assessed in this study, including detailed height information

|  | All FHS Gen 3 exam 3  (n=3521) | **Sample 1**  Participants with valid Actical and Apple Watch data, worn on the same day(s)  (n=523) | **Sample 1, Section A**  Sample 1 participants who had days that fell into Section A, Fig 2 (Actical >30,000 steps, Apple Watch <20,000 steps)  (n=5) |
| --- | --- | --- | --- |
| Age, y | 54.5 | 51.7 | 51.8 |
| Female (%) | 1896 (53.9%) | 298 (57.0%) | 1 (20%) |
| BMI, kg/m^2^ | 28.6 | 28.2 | 25.1 |
| Mobility limitation, n (%) | 703 (20%) | 85 (16.3%) | 0 |
| Height, inches | 66.6 | 66.8 | 69.7 |
| Quintile 1 for height, n (column%) | -- | 20% | 0% |
| Quintile 5 for height, n (column%) | -- | 20% | 40% |

**Supplemental Table 4.** Agreement between steps accumulated on Actical vs. Apple Watch device by participants wearing both devices on the same date (Samples 1 and 2 with additional exclusions and Sample 1X including only days with fewer hours of Apple Watch wear time)

| **Sample** | **Adjusted Linear Regression, β (95%CI)** | **ICC**  **(95% CI)** | **Lin's concordance correlation**  **r (95% CI)** | **Mean difference * steps/day (Bland Altman limits of agreement)** | **Mean % difference ** steps/day (Bland Altman limits of agreement)** | **Percent of Apple Watch days with a step count**  **(person-days or**  **person-hours)** | |
| --- | --- | --- | --- | --- | --- | --- | --- |
|  |  |  |  |  |  | **Within 15% agreement compared to Actical** | **Within 5% agreement compared to Actical** |
| **Sample 1** after excluding all days contributed by participants that fell into Section A, Fig 2  (n=518 participants; n=3188 person-days) | 0.65  (0.63, 0.68) | 0.63  (0.61, 0.65) | 0.63  (0.61, 0.65) | -290  (-8017, 7438) | -7.4%  (-133.4, 118.6) | 30.0% (956) | 10.1% (323) |
| **Sample 1** after excluding days with <1000 or >30,000 steps  (n=512 participants; n=2963 person days) | 0.69  (0.66, 0.71) | 0.68  (0.66, 0.70) | 0.68  (0.66, 0.70) | 40  (-6898, 6979) | 1.8%  (-89.5, 93.1) | 32.2% (954) | 10.9% (323) |
| **Sample 2** after excluding all hours contributed by participants that fell into Section A, Fig 2  (n=18480 person-hours, n=451 participants, 1961 person-days) | 0.96  (0.96, 0.97) | 0.89  (0.89, 0.90) | 0.89  (0.89, 0.90) | 44  (-674, 761) | 18.3%  (-93.7, 130.2) | 27.6% (5097) | 10.2% (1883) |
| **Sample 1X** – only includes days with ≥10h Actical wear time and ≥5 h (but <10h) Apple Watch wear time  (n=19 participants; n=74 person-days) | 0.49 (0.17, 0.81) | 0.22  (-0.06, 0.46) | 0.21  (0.08, 0.33) | -3982  (-11583, 3619) | -71.2%  (-188.5, 46.1) | 12.2% (9) | 5.4% (4) |
| **Sample 1Xb** – Sample X, with additional exclusion for days with <1000 or >30,000 steps  (n=19 participants;  N=65 person-days) | 0.39 (0.15, 0.62) | 0.27 (-0.07, 0.55) | 0.27  (0.13, 0.40) | -3223  (-8981, 2534) | -57.9%  (-155.7, 40.0) | 13.8% (9) | 6.2% (4) |

The adjusted linear regression model includes: age, sex, cohort type, BMI, height, (and the difference in wear time for Sample 1)

* Mean difference is Apple Watch steps minus Actical steps

**Mean % difference was calculated as (Apple Watch steps minus Actical steps)/ (average Apple Watch and Actical steps)

**Supplemental Figure 1.** Example of 24 hours of Actical and Apple Watch data from a participant who was included in Sample 1, but not in Sample 2.

| **Hour** | **Step_actical** | **Step_applewatch** | **Mean HR applewatch** |
| --- | --- | --- | --- |
| 0 | 198 | *NA* | *NA* |
| 1 | 108 | *NA* | *NA* |
| 2 | 0 | *NA* | *NA* |
| 3 | 0 | *NA* | *NA* |
| 4 | 0 | *NA* | *NA* |
| 5 | 891 | *NA* | *NA* |
| 6 | 688 | *NA* | *NA* |
| 7 | 754 | *NA* | *NA* |
| 8 | 454 | *NA* | *NA* |
| 9 | 134 | *NA* | *NA* |
| 10 | 301 | *NA* | 82.3 |
| 11 | 987 | *NA* | 82.4 |
| 12 | 344 | 163 | 81.3 |
| 13 | 550 | *NA* | 82.5 |
| 14 | 321 | *NA* | 80.4 |
| 15 | 113 | 305 | 76.2 |
| 16 | 83 | 100 | 79.3 |
| 17 | 1552 | *NA* | 77.4 |
| 18 | 813 | *NA* | 78.3 |
| 19 | 450 | *NA* | 73.6 |
| 20 | 371 | *NA* | 73.6 |
| 21 | 87 | *NA* | 80.1 |
| 22 | 5 | *NA* | 73.7 |
| 23 | 0 | *NA* | *NA* |

This participant was not included in Sample 2 because they did not have any hours with ≥3 consecutive hours of steps counted by both Actical and Apple Watch. Observe 13 consecutive hours when heart rates were recorded and transmitted by the Apple Watch, but steps were only recorded/transmitted on 3 of those hours.

**Supplemental Figure 2:** The scatteplot shown in Figure 3, but including the single hour that was removed as an outlier (circled in red)


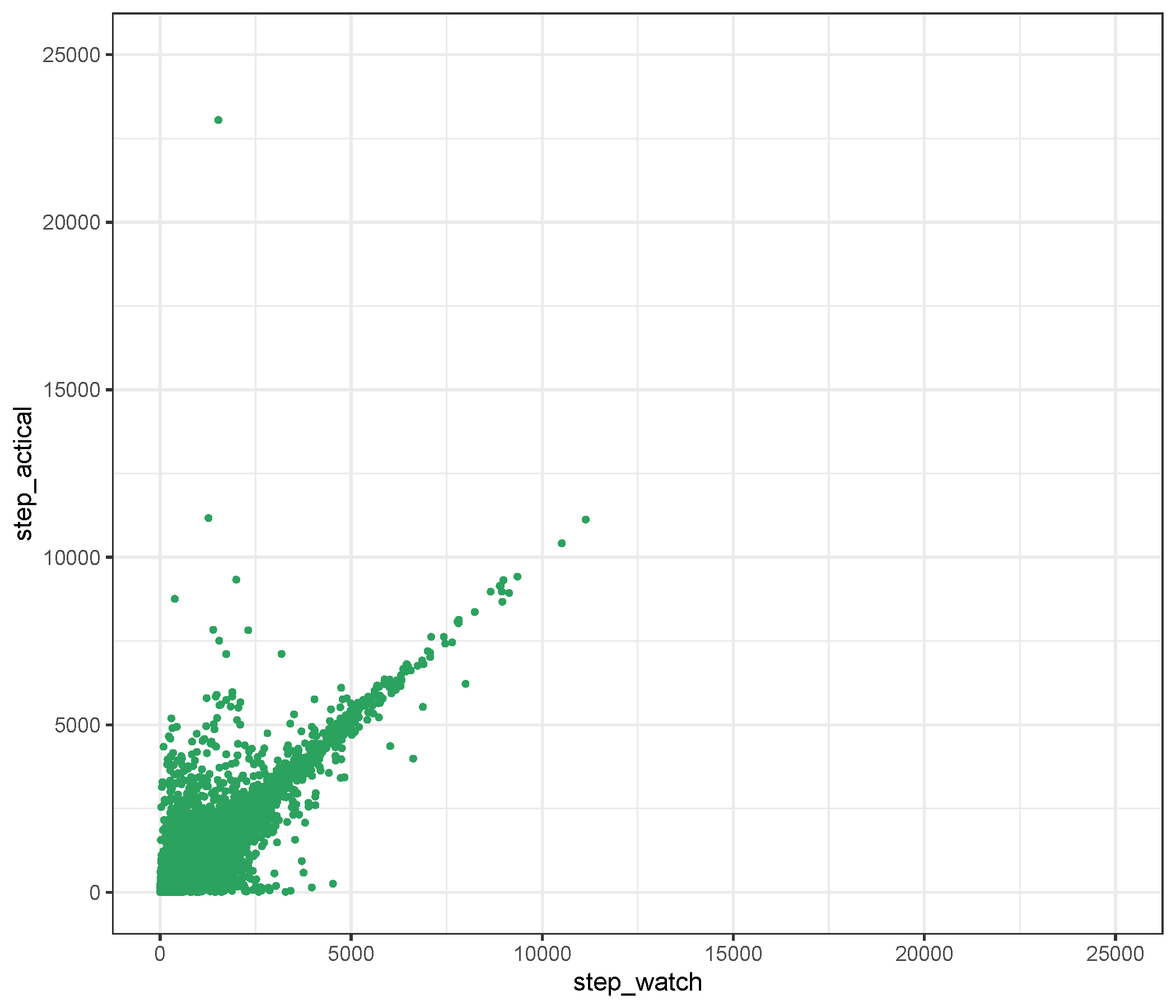


Each point represents data from a single hour (1 person-hour)

**Supplemental Figure 3.** Scatterplot of Apple Watch steps by Actical steps accumulated during hours when both devices were worn, only for participants who fell into section A in Figure 2 (days when Actical counted >30,000 steps and Apple Watch counted <30,000 steps)

**
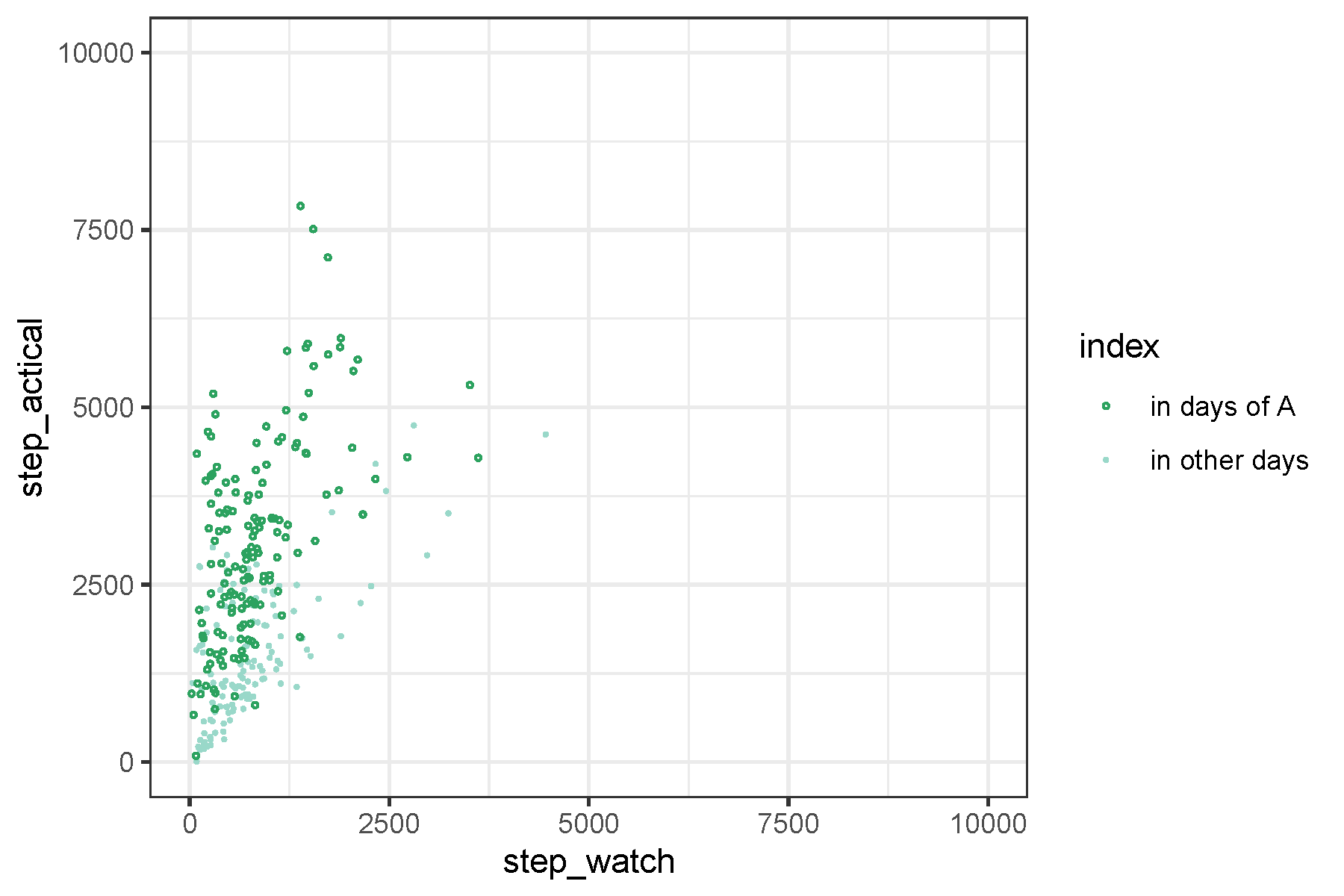

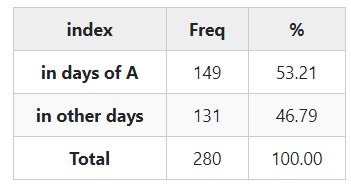
**

A total of 5 participants contributed 17 days to Section A in Figure 2 (Sample 1). In the current figure, dark green circles represent 147 person-hours from these 17 days during which both devices were being worn, using the definition for inclusion in Sample 2. Each datapoint represents one hour on which both devices were worn. The light green circles represent 131 person-hours (during which both devices were worn) from “other days” (not in Section A) from these participants who contributed day(s) to Section A. This scatterplot does not appear to follow a similar pattern as the scatterplot for Figure 3, meaning that there was either something systematically different about how these 5 participants moved or different with the devices compared to the rest of the study sample that led to them contributing days in Section A.

**Supplemental Figure 4:** Scatterplot of Apple Watch steps by Actical steps accumulated during hours when both devices were worn, only for participants who fell into sections B in Figure 2 (days when Apple Watch counted <1000 steps and Actical counted >1000 steps)

**
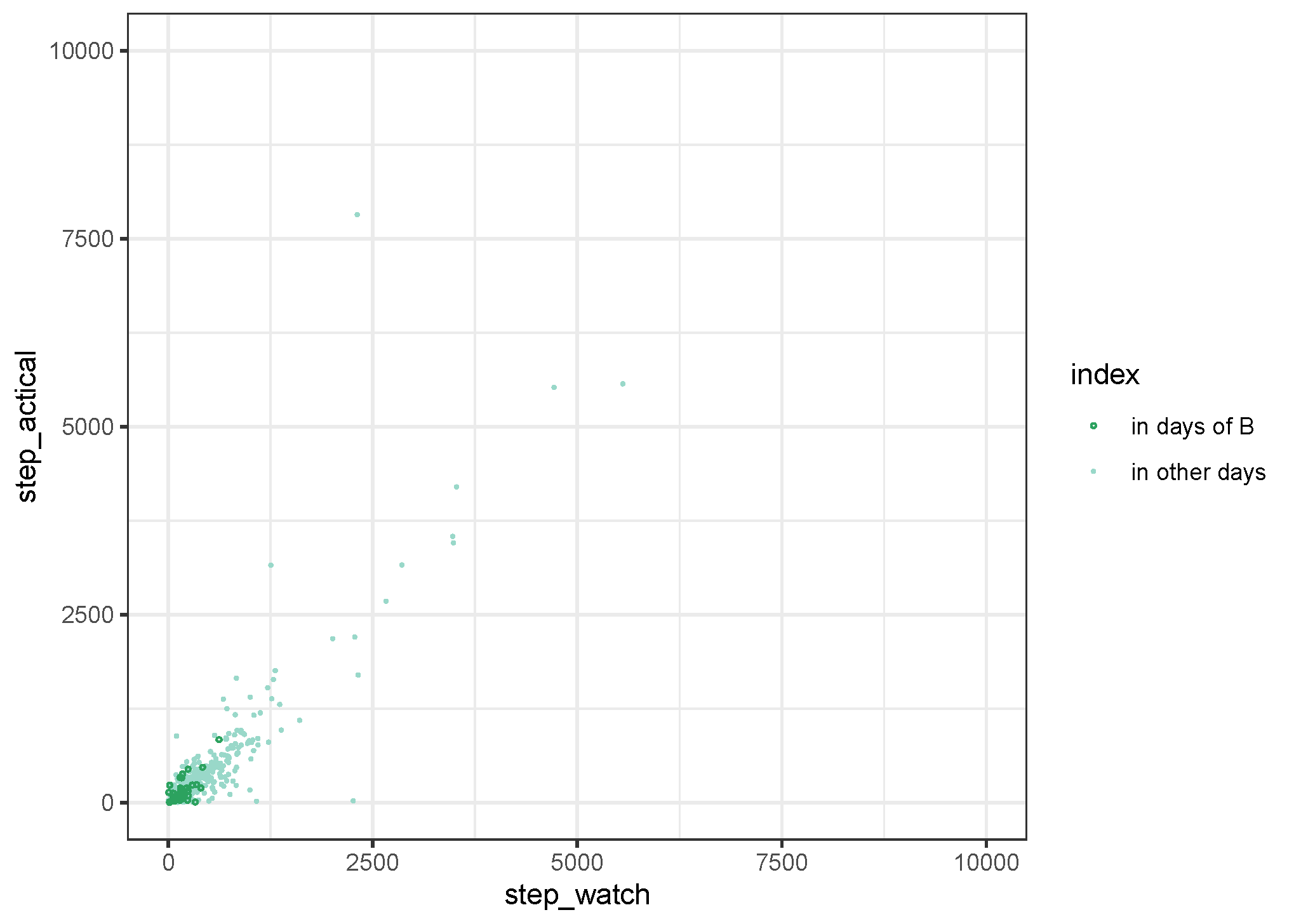

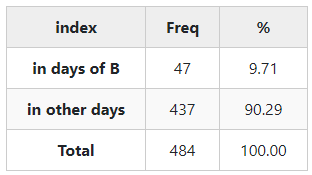
**

A total of 68 participants contributed 205 days to Section B in Figure 2 (Sample 1). In the current figure, dark green circles represent 47 person-hours (from 20 of these participants) during which both devices were being worn, using the definition for inclusion in Sample 2. Most days in Section B did not have any overlapping blocks of hours. Each datapoint represents one hour on which both devices were worn. The light green circles represent 437 person-hours (during which both devices were worn) from “other days” (not in Section B) from 48 of the 68 participants who contributed day(s) to Section B. The scatterplot of “other days” from Section B participants appear to follow a similar pattern as the scatterplot for Figure 3, meaning there was not something systematically different about how these Section B participants moved or their devices that contributed to them having very low Actical step counts. Instead, it is likely that on those Section B days devices were not being worn during the same hours or transmitting/recording step data during the same hours.

**Supplemental Figure 5:** Scatterplot of Apple Watch steps by Actical steps accumulated during hours when both devices were worn, only for participants who fell into sections F in Figure 2 (days when Actical counted <1000 steps and the Apple Watch counted >1000 steps)

**
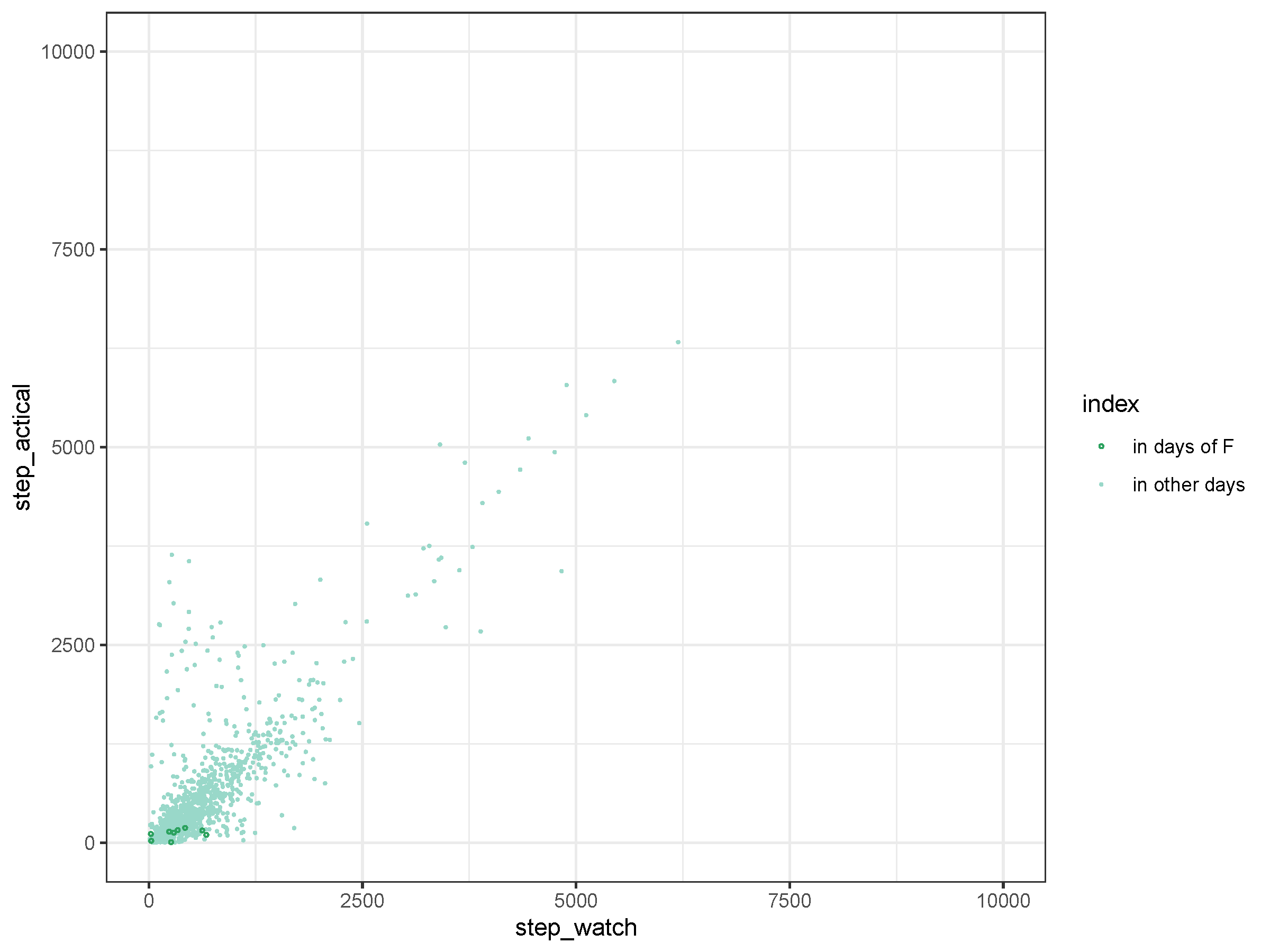

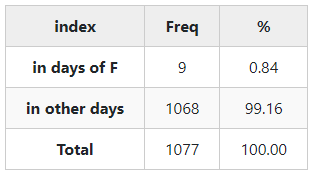
**

A total of 29 participants contributed 31 days to Section F in Figure 2 (Sample 1). In the current figure, dark green circles represent 9 person-hours (from only 2 of these participants) during which both devices were being worn, using the definition for inclusion in Sample 2. Most days in Section F did not have any overlapping blocks of hours. Each datapoint represents one hour on which both devices were worn. The light green circles represent 1068 person-hours (during which both devices were worn) from “other days” (not in Section F) from 24 of the 29 participants who contributed day(s) to Section F. The scatterplot of “other days” from Section F participants appear to follow a similar pattern as the scatterplot for Figure 3, meaning there was not something systematically different about how these Section F participants moved or their devices that contributed to them having very low Actical step counts. Instead, it is likely that on those Section F days devices were not being worn during the same hours or transmitting/recording step data during the same hours.
